# Towards authentically labelled bi-modal PET (SPECT)/MR-probes

**DOI:** 10.1186/2197-7364-1-S1-A79

**Published:** 2014-07-29

**Authors:** Heinrich H Coenen, Martin Buchholz, Ingo Spahn, Christian Vanasschen, Johannes Ermert, Bernd Neumaier

**Affiliations:** Institute of Neuroscience and Medicine, INM-5: Nuclear Chemistry, Research Centre, Jülich, Germany; Institute for Radiochemistry and Experimental Molecular Imaging, Medical Clinics, University of Cologne, Kragujevac, Germany

Application of radiolabelled, existing MRI probes using a suitable reporter group for multimodal PET(SPECT)/MRI imaging is limited due to the required alteration of the molecular structure and thus changing their *in vivo* properties. Radiolabelling of existing MRI contrast agents with PET(SPECT) isotopes of paramagnetic elements offers a simple way to address this issue. Therefore, new routes to the production of SPECT/PET-radionuclides ^147,149^Gd and ^52g^Mn were examined which can be applied for n.c.a. labelling of Gd(III) and Mn(II) MRI contrast agents. Additionally, Mn(II)-based complexes stable for *in vivo* application are to be synthesized.

Reaction cross sections and experimental thick target yields were measured by irradiation of ^nat^Cr or Eu_2_O_3_. Integral yields were calculated from measured excitation functions. A radiochemical separation of Mn from Cr was developed based on cation-exchange chromatography [1].

Cross section data of the ^nat^Eu(d,x) and ^nat^Eu(p,x) reactions were measured up to 70.9 MeV and 44.8 MeV, respectively. Integral yields of up to 177.3 MBq/μAh and 81.6 MBq/μAh for ^nat^Eu(d,x)^147,149^Gd reactions and up to 43.3 MBq/μAh and 61.8 MBq/μAh for ^nat^Eu(p,x)^147,149^Gd reactions, respectively, were calculated. Those were several times higher than for α- or ^3^He induced reactions on highly enriched ^144^Sm [2, 3].

With n.c.a. ^52^Mn, also cross sections of co-produced ^48^V, ^48,49,51^Cr, ^52g^Mn were determined in the energy range of 7.6 to 45 MeV. The production rates of ^52g,m^Mn were measured from 8.2 to 16.9 MeV with up to 13.1 MBq/μAh which was separated from ^nat^Cr by column chromatography.

Production data of the SPECT nuclides ^147,149^Gd and the PET nuclide ^52g^Mn were established. Different to Mn a practical isolation procedure for Gd is still required. Current work focuses on the radiolabelling of stable complexes of manganese (II) with the goal to develop PET/MRI tracers addressing molecular targets.
